# Identification of a novel *TECRL* variant causing type 3 catecholaminergic polymorphic ventricular tachycardia

**DOI:** 10.3389/fped.2025.1549827

**Published:** 2025-05-16

**Authors:** Yun Chen, Jie Shen, Long Chen, Ling Sun

**Affiliations:** ^1^ Department of Cardiology, Children’s Hospital of Soochow University, Suzhou, China; ^2^ Department of Echocardiography, Children’s Hospital of Soochow University, Suzhou, China

**Keywords:** sudden cardiac death, long QT syndrome, catecholaminergic polymorphic ventricular tachycardia, pediatric, *TECRL*, functional analysis

## Abstract

**Background:**

Catecholaminergic polymorphic ventricular tachycardia (CPVT) is a leading cause of sudden cardiac death (SCD) in young patients, characterized by bidirectional or polymorphic ventricular tachycardia often induced by physical exertion or emotional stress.

**Results:**

We analyzed a 12-year-old girl with CPVT who suffered cardiac and respiratory arrest. Clinical data were derived from medical records. Whole-exome sequencing (WES) and Sanger sequencing identified two missense mutations in the trans-2,3-enoyl-CoA reductase-like (*TECRL*) gene (NM_001010874.5: c.587G > A p.Arg196Gln and NM_001010874.5: c.868C > T p.Pro290Ser), potentially pathogenic and associated with type 3 CPVT (CPVT3). Functional studies suggested both mutations could lead to reduced protein expression. We also discovered a novel *TECRL* mutation (NM_001010874.5: c.868C > T p.Pro290Ser). This study further supports the role of *TECRL* as one cause of CPVT.

**Conclusions:**

In this study, functional studies implicate these variants as the cause of CPVT in this patient.

## 1 Introduction

Cardiac arrhythmias are a primary factor contributing to sudden cardiac death among pediatric patients (1). Catecholaminergic polymorphic ventricular tachycardia (CPVT) ranks as one of the frequent triggers of sudden cardiac death in the pediatric and adolescent stages. The typical age at which symptoms first appear falls between 7 and 9 years of age (2). Currently, the estimated global incidence of CPVT is approximately 1 in 10,000, but its actual prevalence is still unknown and may be underestimated (3). Research indicates that SCD resulting from ventricular arrhythmias, even without underlying structural heart issues, often has a genetic origin (1). The condition arises from mutations in genes that code for ion channel or calcium-handling proteins, which can impact the electrical functions of the heart (4). Clinically, it is characterized by the induction of bidirectional or polymorphic ventricular tachycardia during exercise or emotional excitement, which can lead to syncope and even sudden cardiac death. Echocardiography typically reveals a normal cardiac structure (5).

Previously reported individuals harboring disease-causing variants in the trans-2,3-enoyl-CoA reductase-like protein (*TECRL*) gene have exhibited a mixed phenotype of CPVT and long QT syndrome (LQTS), referred to as type 3 CPVT (CPVT3), an autosomal recessive disorder. *TECRL* is a recently identified gene linked to life-threatening inherited heart rhythm disorders that manifest symptoms of both LQTS and CPVT (6). The primary clinical features of CPVT type 3 encompass a range of severe cardiovascular events, including sudden cardiac arrest, a prolonged QT interval, onset during adolescence, and various arrhythmias such as ventricular fibrillation, paroxysmal ventricular tachycardia, polymorphic ventricular tachycardia, bidirectional ventricular tachycardia, and ventricular premature beats. Additionally, patients may experience symptoms like palpitations, syncope, and unexpected death (7). The disease shares similar clinical features with LQTS (6). Children affected by this condition typically remain asymptomatic at rest and exhibit a structurally normal heart. However, adverse events such as malignant arrhythmias, syncope, and cardiac arrest are commonly triggered by physical exertion or emotional stressors (8). The condition exhibits a substantial rate of fatality and is prone to misdiagnosis, requiring close attention from clinicians to avoid missed or incorrect diagnoses.

Six genes with mutations have been linked to CPVT, exhibiting inheritance patterns ranging from autosomal dominant (*RyR2, KCNJ2, CALM1*, and *CALM2*) to autosomal recessive (calcium sequestration protein *CASQ2* and calcium release complex *TRDN*) (9, 10). Approximately 65% of cases of CPVT are attributed to autosomal dominant mutations in the *RyR2* gene, which encodes the cardiac ryanodine receptor. The remaining cases are caused by less common autosomal recessive mutations in genes that code for calcium storage proteins. This distinction in mutation types provides a clearer understanding of the genetic basis for CPVT and informs genetic counseling and diagnostic strategies (11), *CASQ2* variants account for 2%–5% of all CPVT cases (12).

Homozygous variants of Arg196Gln in the *TECRL* gene were found in two Francophone Canadian CPVT probands, and a homozygous splice site mutation (c.331 + 1G > A) within the *TECRL* gene was found in a Sudanese family affected by CPVT. *TECRL* gene mutations (Arg196Gln and c.331 + 1G > A splice site mutation) were initially described as being associated with CPVT (6). Compared to other pathogenic genes, *TECRL* gene mutations are very rare in CPVT (13). Xiao et al.'s research revealed a compound heterozygous mutation in the *TECRL* gene (Arg196Gln and c.918 + 3T > G splice site mutation) in a 13-year-old male patient with CPVT. Through Sanger sequencing, they confirmed that the c.918 + 3T > G mutation could impact the alternative splicing of *TECRL*, indicating *TECRL* as a newly identified gene linked to CPVT (13).

In the present study, we detected two mutations of *TECRL* gene (mut1/m1: NM_001010874.5: c.587G > A p.Arg196Gln and mut2/m2:NM_001010874.5: c.868C > T p.Pro290Ser) in a 12-year-old girl with CPVT and LQTS who had cardiac and respiratory arrest using WES. Bioinformatic analyses revealed that 2 variants were potentially pathogenic and related to CPVT3, and functional studies indicated that both variants could lead to reduced protein expression. These findings support their role in disease. A novel *TECRL* mutation (NM_001010874.5: c.868C > T p.Pro290Ser) was detected within CPVT. Our research broadens the molecular landscape of CPVT.

## 2 Materials and methods

### 2.1 Human subjects and clinical data collection

A 12-year-old girl, the index case, was brought to the hospital because she experienced sudden cardiopulmonary arrest without a clear trigger while playing at home. We collected comprehensive clinical information, including the onset of signs and symptoms, disease change, treatments, 12-lead ECG, Holter, Echocardiography, cranial CT and myocardium markers tests. The patient at the center of this study, was treated at the Children's Hospital of Soochow University in February 2023.

We acquired informed consent from the participant's guardian and received approval from the Ethics Committee of Soochow University Children's Hospital. The study was conducted in accordance with the principles and guidelines set forth in the Declaration of Helsinki.

### 2.2 Whole-exome sequencing (WES) and bioinformatic analyses

This study employed high-throughput sequencing to analyze the complete exome. Briefly, 1 microgram of genomic DNA was extracted from a 200 microliter sample of peripheral blood using the Qiagen DNA Blood Midi/Mini Kit (Qiagen GmbH, Hilden, Germany), adhering to the kit's instructions. Subsequently, library preparation was carried out with the Nano WES Human Exome V2.0 kit (Berry Genomics, China), as recommended by the manufacturer. The sequencing was performed on the Novaseq6000 platform (Illumina, San Diego, USA). The generated sequencing reads were aligned to the human reference genome (hg19/GRCh37) utilizing the Burrows-Wheeler Aligner tool, and duplicate PCR duplicates were subsequently filtered out with the help of Picard version 1.57. For variant detection, Berry Genomics’ Verita Trekker® system and the GATK software (Broad Institute) were utilized. Variant annotation and its interpretation were handled by ANNOVAR and the Enliven® Variant Annotation Interpretation System, both authorized by Berry Genomics. Population frequency filtering was applied using databases such as the 1,000 Genomes Project, Exome Aggregation Consortium, and Genome Aggregation Database, considering only variants with frequencies below 1 in 1,000. The potential pathogenicity or harmfulness of the variants was assessed using SIFT, PolyPhen-2, and CADD, while their impact on splicing was predicted with Varseak, Human Splicing Finder, and SpliceAI. To enhance clinical diagnostic utility, known pathogenic variants from the Human Gene Mutation Database (HGMD)and ClinVar (March 2023 update) were also considered for further analysis. Variants were categorized as “pathogenic,” “likely pathogenic,” “of uncertain significance,” “likely benign,” or “benign,” based on the American College of Medical Genetics and Genomics (ACMG) guidelines for the interpretation of genetic variants (14). Finally, suspected SNVs and Indels were confirmed through Sanger sequencing.

### 2.3 Construction of plasmids and transfection protocol

The complete cDNA sequence for *TECRL* was inserted into the pcDNA3.1 vector for expression. The pcDNA3.1 plasmid was acquired from Bioeagle Biotech Co., Ltd., based in Wuhan, China. The plasmid was engineered to incorporate restriction sites and the complete *TECRL* sequence using specific primers (pcDNA3.1-Flag-EFla-copGFP-TECRL-EcoRI-F/R), resulting in the creation of the recombinant vector pcDNA3.1-Flag-EFla-copGFP-wt (wild type). Primers for two mutant variants, designated TECRL-mut1-F/R and TECRL-mut2-F/R, were synthesized. Fragment 1 was amplified by using pcDNA3.1-Flag-EFla-copGFP-TECRL-EcoRI-F and TECRL-mut1-R as primers, and fragment 2 was amplified by using TECRL-mut1-F and pcDNA3.1-Flag-N-TECRL-NotI-R as primers. With a 1:1 combination of Fragment 1 and Fragment 2 as the template, the pcDNA3.1-Flag-EFla-copGFP-mut1 fragment, harboring the mutation c.587G > A:p.Arg196Gln, was generated through overlap extension PCR using the primers pcDNA3.1-Flag-EFla-copGFP-TECRL-EcoRI-F and pcDNA3.1-Flag-EFla-copGFP-TECRL-NotI-R. Using the same experimental method, the mutation c.868C > T:p.Pro290Ser were introduced into the above-mentioned recombination vectors. The recombinant vectors pcDNA3.1-Flag-EFla-copGFP-mut1 and pcDNA3.1-Flag-EFla-copGFP-mut2 were constructed respectively. The sequences of the primers employed in the experiments are provided in Table 1. The integrity of the two recombinant plasmids was confirmed by sequencing. Human embryonic kidney 293 T cells were cultured in DMEM medium enriched with 10% fetal bovine serum (from Gibco, Grand Island, NY, USA) at a temperature of 37°C and under a 5% CO_2_ environment. The 293 T cells were transiently transfected with both the wild-type and mutant plasmids using Lipofectamine 3,000 reagent (from Invitrogen, Carlsbad, CA, USA) in accordance with the instructions provided by the manufacturer. Following a 48-hour transfection and culture period, total RNA and proteins were isolated and analyzed using real-time PCR (qPCR) and western blot techniques.

**Table 1:** The primers used in construction of the plasmid, point mutation, and qPCR.

Primers	Sequences (5′–3′)
pcDNA3.1-Flag-EFla-copGFP-TECRL-EcoRI-F	ggtggaattcgATGTTCAAAAGGCACAAGTC
pcDNA3.1-Flag-EFla-copGFP-TECRL-NotI-R	tcgagcggccgcTTACAATATGAATGGAATCA
TECRL-mut1-F	GTATACACTACATCCAATACCTTTTGGAAAC
TECRL-mut1-R	GTTTCCAAAAGGTATTGGATGTAGTGTATAC
TECRL-mut2-F	AGTCCAAATTATAACTCCTTCACATGGATGT
TECRL-mut2-R	ACATCCATGTGAAGGAGTTATAATTTGGACT
TECRL-qPCR-F	GGGCCCTCTAAGACCAACTC
TECRL-qPCR-R	GGGTACCACTTTGGACATGC

### 2.4 The quantitative Rt-PCR

RT-PCR was used to evaluate the mRNA expression levels of *TECRL*. Briefly, after transient transfection of 293 T cells with both the wild-type and mutant plasmids, total cellular RNA was extracted using the protocols provided by the manufacturer (Takara, Japan). The specific primer pairs used for *TECRL* amplification (forward and reverse, TECRL-qPCR-F/R) are detailed in Table 1.

### 2.5 Western blotting (WB)

Protein extracts were collected, and their concentrations were quantified with the aid of the BCA protein assay kit. (Yeasen Biotechnology Shanghai, China). The protein samples were separated using a 10% polyacrylamide gel with sodium dodecyl sulfate and then electrotransferred onto a polyvinylidene fluoride (PVDF) membrane (Millipore, Bedford, MA, USA). The membrane was subsequently incubated with primary antibodies targeting Flag (Mabnus, Shanghai, China, product GS20002), GAPDH (Cell Signaling Technology, Danvers, MA, USA, product 2118S), and copGFP (Oasis, Hangzhou, China, product OB-PRT011) at a temperature of 4°C for overnight periods. Blots were incubated with secondary antibodies against HRP-conjugated Affinipure Goat Anti-Mouse IgG(H + L), (Proteintech, SA00001-1), HRP-conjugated Affinipure Goat Anti-Rabbit IgG(H + L), (Proteintech, SA00001-2), HRP-Goat Anti-Rat IgG (H + L), (Diaan, Q1003) for another hour at room temperature. Once washed, the blots were detected using a chemiluminescent reagent, followed by analysis with image J software. To validate the findings of this study, we conducted three separate experiments in 293 T cells, employing the identical experimental approach as previously described.

## 3 Result

### 3.1 Clinical findings

**Figure 1:**
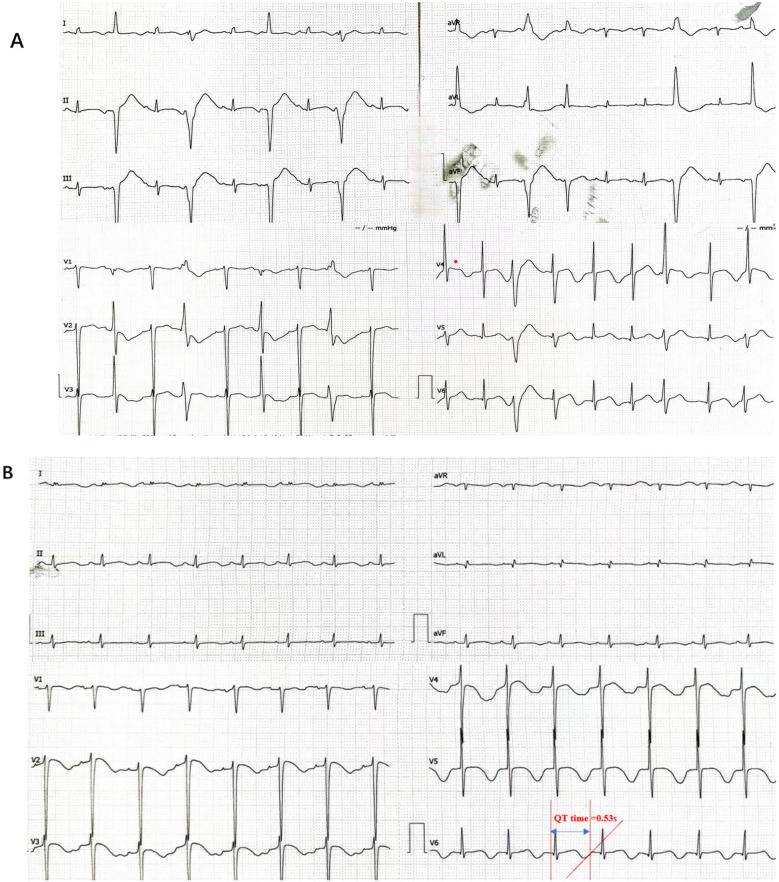
The patient's electrocardiogram indicated arrhythmia. **(A)** The electrocardiogram suggested frequent ventricular premature beats, some occurring in pairs. **(B)** Prolonged QT interval:QT time = 0.53 s (QTc:0.65 s).

A 12-year-old girl experienced sudden cardiopulmonary arrest without a clear trigger while playing at home. She was taken to the local hospital's emergency department where endotracheal intubation, cardiopulmonary resuscitation, and defibrillation were performed, resulting in the restoration of sinus rhythm. However, she frequently developed ventricular fibrillation, which required additional defibrillation and treatment with amiodarone infusion. She was then transferred to our hospital for inpatient treatment. The child was in a coma and received mechanical ventilation. The 12-lead ECG showed frequent ventricular premature beats, some in pairs, and a prolonged QT interval (QTc: 0.65 s). Although we did observe multiple episodes of ventricular tachycardia during cardiac monitoring, we unfortunately failed to capture these events on standard 12-lead ECG due to their transient nature. Echocardiography revealed normal cardiac structure and function. The cranial CT was normal. The levels of creatine kinase MB (CKMB) and high-sensitivity troponin T were elevated (considering the damage after defibrillation and myocardial ischemia). During the child's hospitalization, the electrocardiographic monitoring showed frequent ventricular premature beats and episodes of short-lasting ventricular tachycardia. The Holter monitor revealed sinus rhythm, occasional sinus arrests, ventricular premature beats, and prolonged QTc interval (Figure 1). The child's birth history, personal history, and past medical history were unremarkable. The family medical history showed that the child's mother had a syncopal episode at the age of 10, but there are no medical records or treatment information available. The parents and a 5-year-old brother were healthy, and their resting ECGs showed sinus rhythm. In light of the above clinical findings and medical history, the patient was diagnosed with CPVT and treated with oral mexiletine and propranolol. The electrocardiogram no longer suggested ventricular tachycardia attacks, but ventricular premature beats persisted. The child experienced cardiorespiratory arrest but achieved return of spontaneous circulation (ROSC) following aggressive resuscitation. Although sinus rhythm was restored, the patient remained in a comatose state (GCS:4 T) and was managed with tracheostomy and mechanical ventilation. After initiating antiarrhythmic therapy no recurrent episodes of ventricular tachycardia or subsequent cardiorespiratory arrests were observed. Given the stabilized cardiac condition, the patient's family has requested transfer to an external facility for hyperbaric oxygen therapy (HBOT) and without implantable cardioverter-defibrillator (ICD) placement.

### 3.2 Pathogenicity classification of the genetic mutation

**Figure 2:**
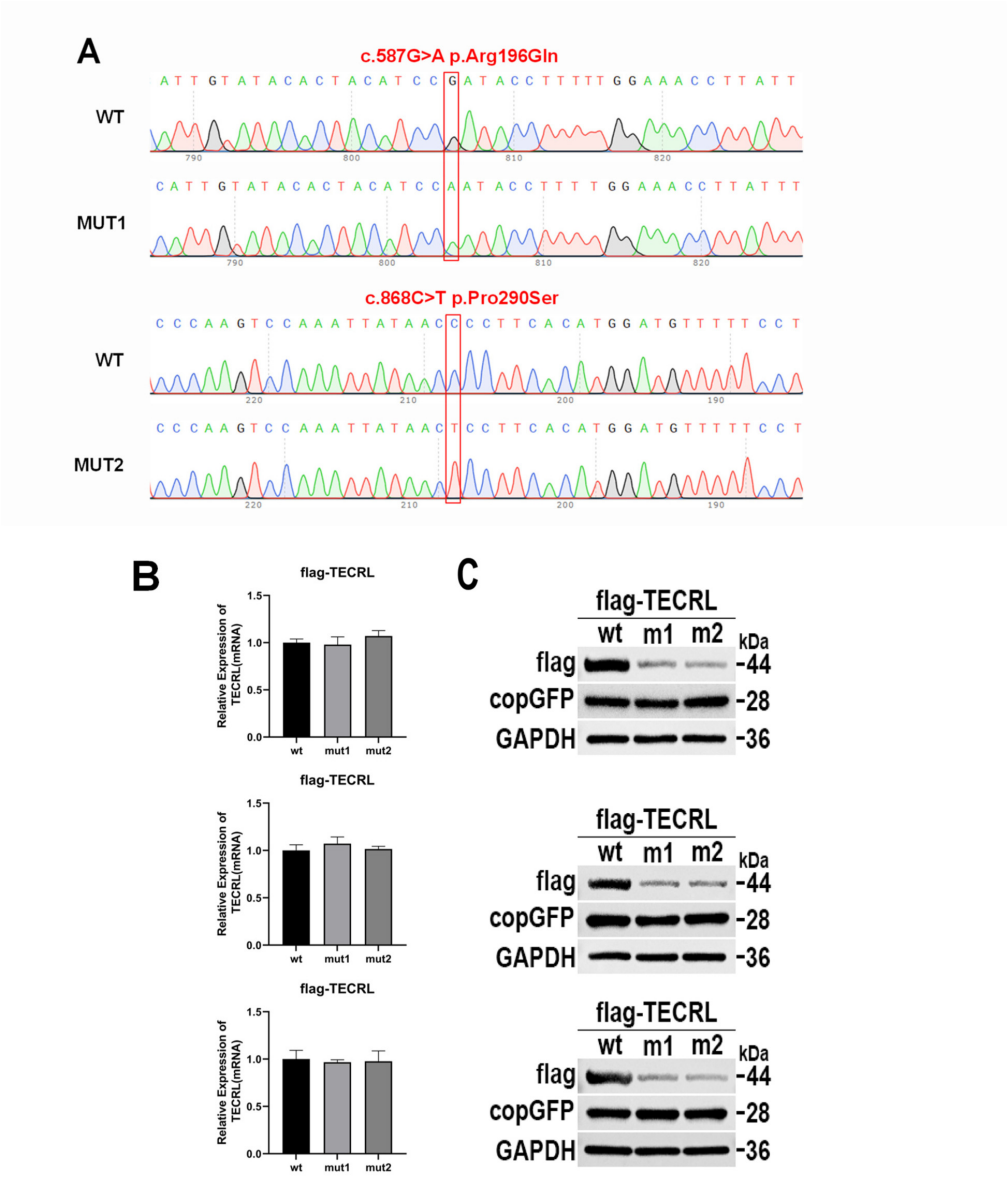
The mutation c.587G > A:p.Arg196Gln and c.868C > T:p.Pro290Ser affects *TECRL* expression. wt, wild type *TECRL*; m1/mut1: c.587G > A:p.Arg196Gln;m2/mut2: c.868C > T:p.Pro290Ser. **(A)** Sequencing results showed that mutation c.587G > A:p.Arg196Gln and c.868C > T:p.Pro290Ser were successfully introduced; **(B)** mRNA expression detected by qPCR; **(C)** *TECRL* protein expression results by WB; **(D)** Bar graph of gray-scale scan statistics of WB results; **(E)** Gel electrophoresis image of quantitative RT-PCR results.

In the proband, WES revealed a compound heterozygous mutation in the *TECRL* gene (NM_001010874.5), characterized by the variants c.587G > A p.Arg196Gln and c.868C > T p.Pro290Ser) (Figure 2). Subsequent validation through Sanger sequencing confirmed that the c.587G > A p.Arg196Gln variant was inherited paternally, whereas the c.868C > T p.Pro290Ser variant was maternally inherited. Notably, the c.587G > A p.Arg196Gln mutation has been previously documented in the literature, whereas the c.868C > T p.Pro290Ser variant represents a novel finding.

**Figure:**
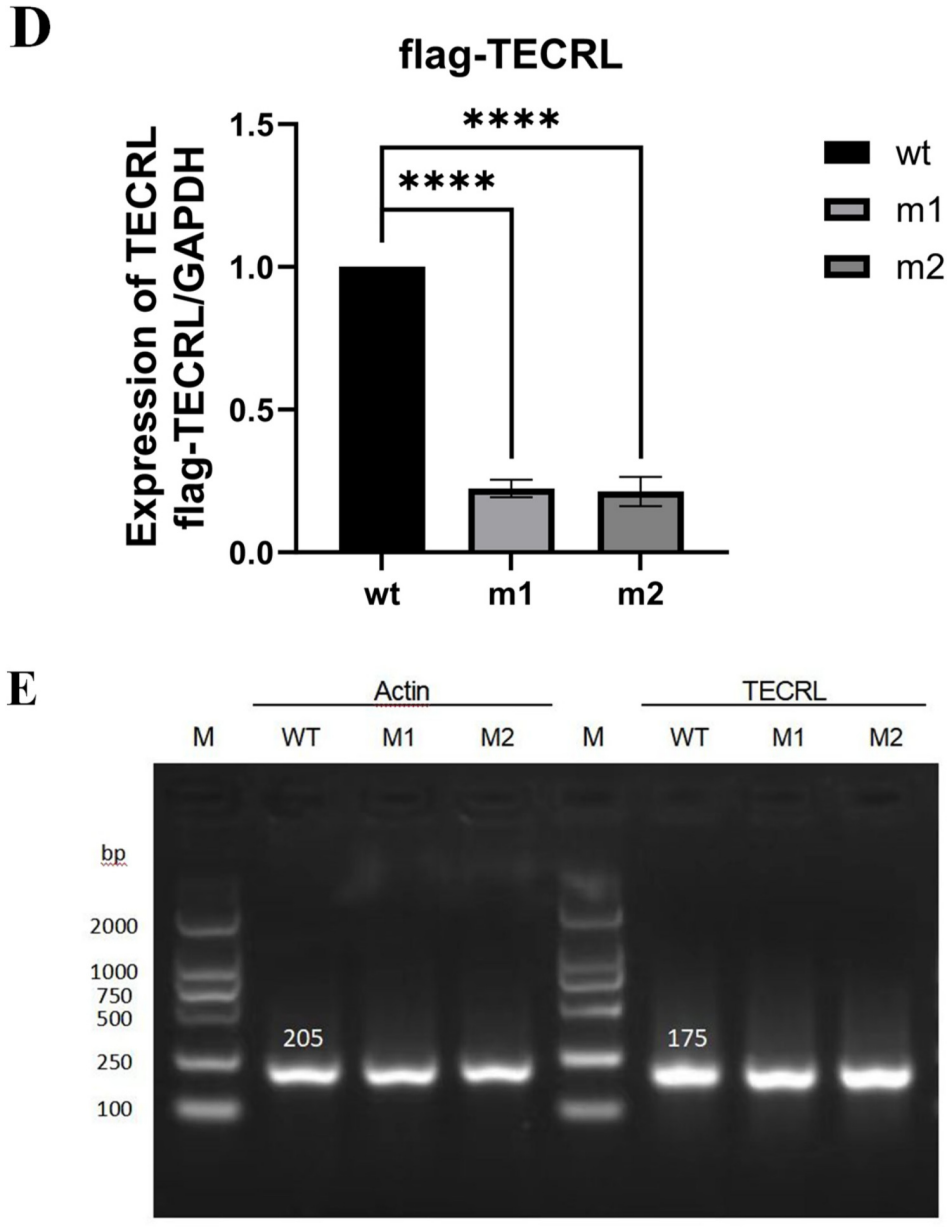


In adherence to the guidelines established by the ACMG (14) and the recommendations provided by the ClinGen Sequence Variant Interpretation (SVI) expert panel (15–17), along with the screening of gene variants associated with clinical phenotypes or diseases in public databases such as the Human Phenotype Ontology (HPO), Online Mendelian Inheritance in Man (OMIM), and the Genome Health Resource (GHR), the mutations c.587G > A and c.868C > T in the *TECRL* gene are proposed to be potential pathogenic variants. The *TECRL*: NM_001010874.5:exon6: c.587G > A p.Arg196Gln mutation is classified as likely pathogenic (PM3_Strong + PM2). This variant has been reported in homozygous form in multiple patients and as a pathogenic mutation in the trans position in one patient (6, 13) (PM3_Strong). It is absent in the 1,000 Genomes and Shenzhou Genome databases. The allele frequencies in the Exome Aggregation Consortium (ExAC) and the Genome Aggregation Database (gnomAD) are 1.65046460578653e-05 and 6.57549e-05, respectively. This known variant is categorized as a variant of limited clinical significance (LP) in the ClinVar database and as a disease-causing variant (DM) in the HGMD (6, 13) (PM2). The *TECRL*: NM_001010874.5: c.868C > T p.Pro290Ser mutation is considered “VUS—favor pathogenic” according to ACMG criterion with the following evidence:PM2(2 points): It is not found in the 1,000 Genomes Project dataset or the Shenzhou Genome Database. The frequencies in ExAC and gnomAD are 8.29338685332316e-06 and 5.59597e-05, respectively. PM3 (2 points): c.587G > A p.Arg196Gln mutation is likely pathogenic, the variant is in compound heterozygosity with the c.587G > A variant, and the proband is phenotype-positive. PS3_supporting (1 point): our functional studies suggested both mutations could lead to reduced protein expression. (Figure 2C), per Brnich et al. (18) guidelines. The total score of 5 points now classifies this variant as “VUS—favor pathogenic” (PM2 + PM3+ PS3_supporting).

In silico prediction tools, including Varseak, Human Splicing Finder, and SpliceAI, suggest that neither mutation significantly impacts splicing. However, as both mutations are located in the middle of the exon, the actual impact on splicing may differ from the predicted results. Public database queries indicate that mutations in *TECRL* (OMIM:617242) can lead to CPVT3 (6)(OMIM:614021).

### 3.3 *In vitro* functional analysis of the *TECRL* variants

To investigate the impact of the variants c.587G > A p.Arg196Gln and c.868C > T p.Pro290Ser on *TECRL* expression in cellular models, we first generated a wild-type *TECRL* expression vector. Subsequently, we employed site-directed mutagenesis to create a *TECRL* mutant expression vector harboring the aforementioned variants. As depicted in Figure 2A, the introduction of the c.587G > A p.Arg196Gln and c.868C > T p.Pro290Ser mutations was successfully confirmed. Regarding mRNA expression of GAPDH and copGFP, as illustrated in Figures 2B,E, there was no notable distinction in mRNA expression levels between the cells transfected with the wild-type plasmid and those transfected with the mutant plasmid, suggesting the mutations do not alter transcription in this plasmid system. WB analysis was performed to evaluate the protein expression levels (Figure 2C,D). When compared to the wild-type *TECRL* protein (44 kDa), both the c.587G > A p.Arg196Gln and c.868C > T p.Pro290Ser variants were missense mutations that at least some of the protein was likely fully processed into a full-length protein. It is possible that some prematurely terminated protein was present but that it was degraded quickly.

## 4 Discussion

Arrhythmogenic inherited diseases (IADs) represent a frequent cause of SCD in pediatric and adolescent populations. IADs can be categorized according to the presence or lack of structural anomalies in the cardiac system. The group without structural heart defects encompasses channelopathies like LQTS, Brugada Syndrome, and CPVT, mainly triggered by genetic mutations in ion channel or calcium-handling protein genes, which impact cardiac electrical function (4, 19).

The etiology of many IADs remains unclear. LQTS is a hereditary arrhythmic disorder distinguished by a prolonged QT interval on a 12-lead electrocardiogram, episodes of torsades de pointes, and an increased risk of unexpected cardiac arrest (20, 21). CPVT is a familial arrhythmia condition marked by ventricular tachycardia triggered by adrenergic stimulation.

Studies suggest that CPVT3 is an autosomal recessive condition resulting from homozygous mutations or intricate heterozygous disease-causing variants in the *TECRL* gene, presenting with a clinical picture that includes features of both CPVT and LQTS (6).

The *TECRL* gene is situated in the 4q13 region of the chromosome and is composed of 12 exons. The protein encoded by this gene comprises 363 amino acids and is mainly found in the sarcoplasmic reticulum of cardiac and skeletal muscle cells, playing a role in the control of lipid metabolism. Norepinephrine also increases the propensity for arrhythmias due to delayed afterdepolarizations in homozygous iPSC-derived cardiomyocytes (13, 22). The *TECRL* protein plays a critical role in fatty acid and lipid metabolism (23, 24).Gene ontology analysis confirms its catalytic function in fatty acid oxidation-reduction reactions. In cardiac tissue, free fatty acids serve as the primary energy substrate during resting states. Abnormal fatty acid oxidation is directly associated with arrhythmogenesis, which may explain how *TECRL* mutations contribute to the development of cardiac arrhythmias.

In 2016, Devalla et al. (6) used an iPSC cell model to compare the homozygous Arg196Gln mutation, heterozygous mutation, and wild-type of the *TECRL* gene. They found that *TECRL* gene mutations (homozygous and heterozygous) led to increased diastolic Ca2 + concentrations, reduced amplitude of calcium transients, and a substantial reduction in intracellular calcium reserves in the sarcoplasmic reticulum. Additionally, the activity of the calcium pumps *SERCA2a* and *NCX* was reduced in the mutant cells, resulting in a significantly prolonged duration of calcium transient decay and an extended action potential duration. This suggests that the gene mutation may lead to an overlap of clinical manifestations between CPVT and LQTS, although the specific mechanism remains unclear. In 2019, Chinese scholars Xie et al. (13) reported a case of CPVT caused by a *TECRL* gene mutation, with the mutation site also being Arg196Gln. To date, there is a lack of in-depth functional studies on *TECRL* gene mutations.

In the present investigation, WES uncovered a 12-year-old female patient who experienced sudden cardiac arrest, carried a compound heterozygous mutation in the *TECRL* gene: c.587G > A p.Arg196Gln and c.868C > T p.Pro290Ser. Although bioinformatics prediction tools have indicated that these variants are likely pathogenic, the clinical significance of these mutations remains to be fully established. To further delineate the clinical relevance of these variants, further functional research is warranted, including assessments of pathogenicity within cellular or animal-based disease models, as recommended by the ACMG guidelines (14). Consequently, to ascertain the pathogenicity of the identified variant, we conducted a series of functional assays for validation.

Further functional experiments, including gene amplification and protein expression assays, confirmed that both mutations can directly lead to reduced protein expression. It is speculated that both loci are pathogenic genes for CPVT. However, these mutations did not significantly affect mRNA transcription or protein synthesis, leading to the hypothesis that they may act through the protein degradation pathway, which requires further investigation.

The c.868C > T p.Pro290Ser mutation is of particular interest as it represents a newly identified pathogenic site within the *TECRL* gene, expanding the spectrum of known mutations associated with this gene. It is worth noting that both the novel missense variant discovered (c.868C > T p.Pro290Ser) and the previously documented variant (Arg196Gln) appear to result in either increased degradation or decreased translation efficiency. In terms of clinical manifestations and age at onset, the compound heterozygous variants observed in this study bear similarities to a previously documented homozygous case. This convergence implies a consistent phenotype in the presentation of the disease (6, 25).

In this study, we identified compound heterozygous *TECRL* mutations (c.587G > A p.Arg196Gln and c.868C > T p.Pro290Ser) in the proband with asymptomatic heterozygous parents, supporting an autosomal recessive inheritance pattern. However, further research is needed to clarify its penetrance and pathogenic mechanisms. Clinical management should integrate genetic testing, family analysis, and functional validation for comprehensive assessment. Initial *in vitro* studies demonstrated significantly reduced protein expression, maybe through increased degradation or decreased translation efficiency. To definitively establish pathogenicity and mechanistic insights, we plan to conduct the more experiments in the future: (1) generating patient-derived iPSC-CMs to examine electrophysiological and calcium handling abnormalities; (2) developing knock-in animal models to assess exercise-induced arrhythmogenesis. These investigations will validate the genotype-phenotype correlation, elucidate the molecular pathophysiology of *TECRL*-deficient CPVT, and provide platforms for developing targeted therapies, while also improving genetic counseling for affected families.

## 5 Conclusion

We have uncovered a new mutation in the *TECRL* gene, c.868C > T p.Pro290Ser, in a young girl presenting with CPVT. Preliminary functional studies implicate these variants in the patient's disease.Genetic testing is regarded as the most reliable method for diagnosing CPVT, and accordingly, we advocate for the inclusion of *TECRL* gene screening in individuals who are clinically suspected of having CPVT, LQTS, or exercise-induced ventricular arrhythmias, as well as in cases of SCD of unknown etiology. This screening should be performed using WES or Whole Genome Sequencing (WGS), tailored to the specific needs of the individual or family under consideration. The genetic insights gained from such testing can inform clinical treatment and prognostic management strategies, ensuring that patients receive appropriate care. Furthermore, these findings can also guide reproductive decisions, aiming to mitigate or prevent the transmission of these potentially life-threatening conditions. Ultimately, our results underscore the utility of WES as a powerful tool for elucidating the genetic underpinnings of SCD, thereby contributing to more accurate diagnoses and improved patient outcomes.

## Data Availability

The datasets presented in this study can be found in online repositories. The names of the repository/repositories and accession number(s) can be found in the article/Supplementary Material.
