# Supplementary material for: Identification of a novel TECRL variant causing type 3 catecholaminergic polymorphic ventricular tachycardia
Source: Front Pediatr. 2025 May 16;13:1549827. doi: 10.3389/fped.2025.1549827 (PMC12122765; doi:10.3389/fped.2025.1549827)
Supplement: Supplementary file 1 [file Datasheet1.docx]

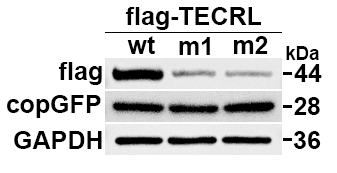


**TECRL-1**

**
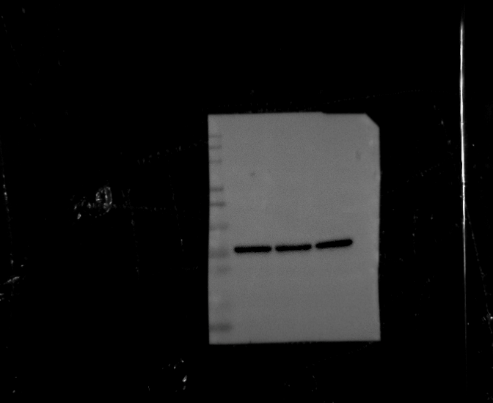
**

**TECRL-COPGFP**

**
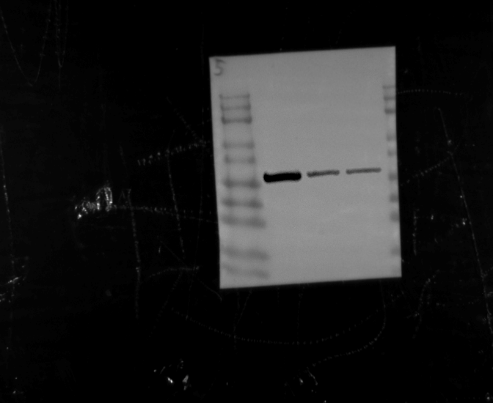
**

**TECRL-FLAG**

**
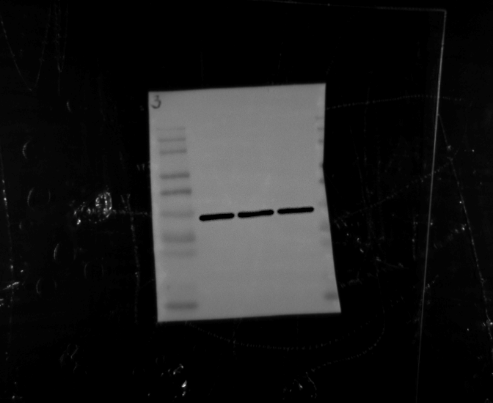
**

**TECRL-GAPDH**

**
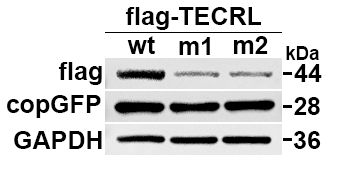
**

**TECRL-2**

**
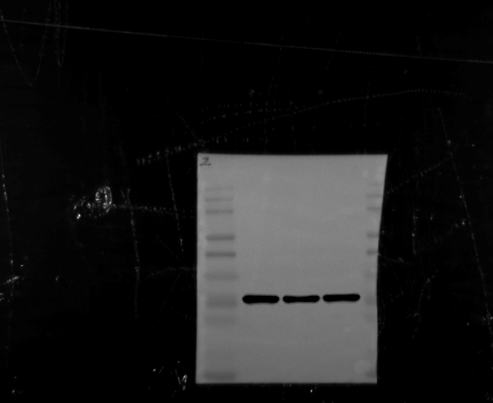
**

**TECRL-COPGFP**

**
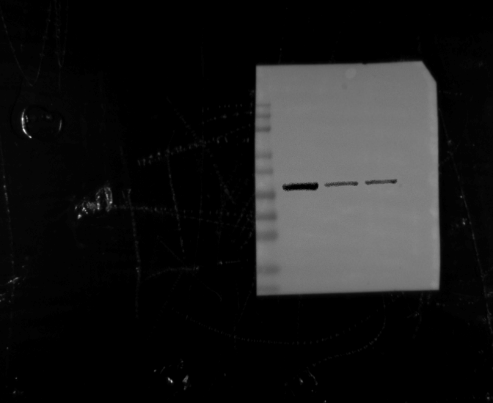
**

**TECRL-FLAG**

**
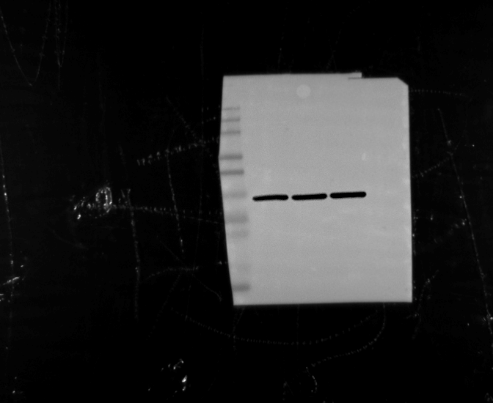
**

**TECRL-GAPDH**

**
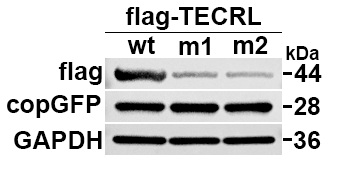
**

**TECRL-3**

**
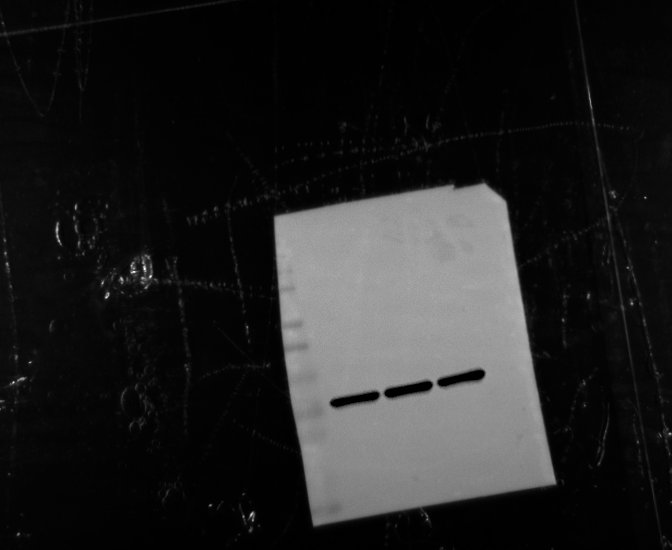
**

**TECRL-COPGFP**

**
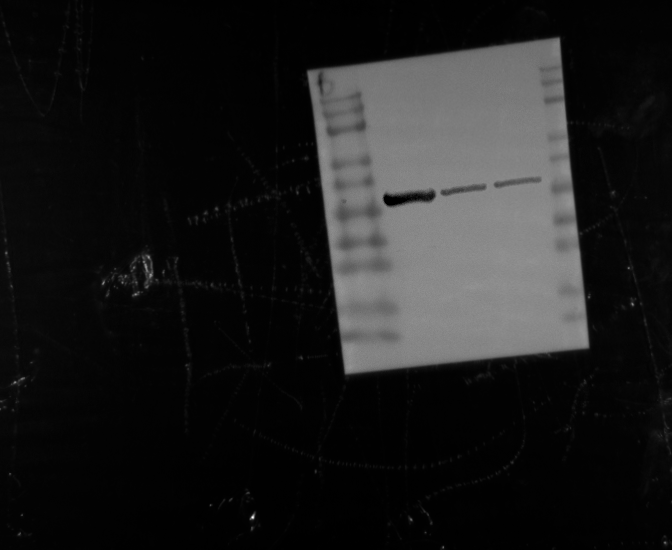
**

**TECRL-FLAG**

**
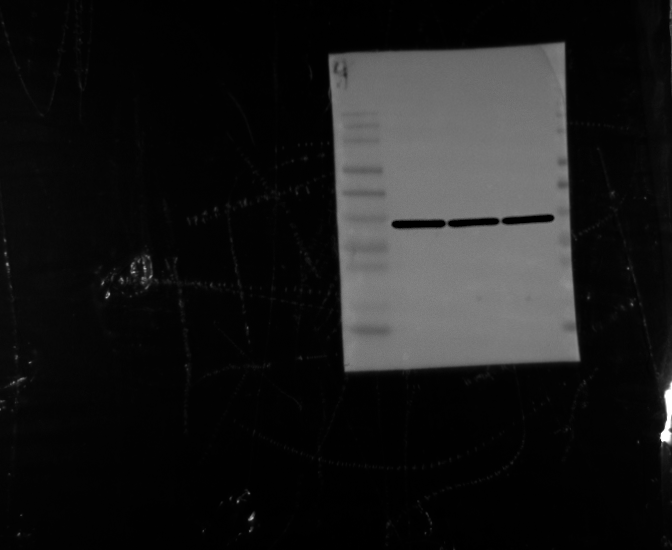
**

**TECRL-GAPDH**
